# Asymptomatic Deep Vein Thrombosis in a Patient with Major Depressive Disorder

**DOI:** 10.1155/2012/261251

**Published:** 2012-09-29

**Authors:** Takuto Ishida, Takeshi Katagiri, Hiroyuki Uchida, Takefumi Suzuki, Koichiro Watanabe, Masaru Mimura

**Affiliations:** ^1^Department of Psychiatry, Sakuragaoka Memorial Hospital, 1-1-1 Renkouji, Tama-shi, Tokyo 206-0021, Japan; ^2^Department of Neuropsychiatry, Keio University School of Medicine, 35 Shinanomachi, Shinjuku-ku, Tokyo 160-8582, Japan; ^3^Geriatric Mental Health Program, Centre for Addiction and Mental Health, Queen Street Site, 1001 Queen Street West, Toronto, ON, Canada M6J 1H4

## Abstract

Pulmonary embolism is a serious, life-threatening condition and most commonly derives from deep vein thrombosis of the lower extremities. Once deep vein thrombosis (DVT) reaches a proximal vein (i.e., popliteal vein or higher), pulmonary embolism reportedly occurs in up to 50% of patients. *Case Presentation*. We report on an inpatient with major depressive disorder in a catatonic state in whom an asymptomatic proximal deep vein thrombosis of 11 × 70 mm was detected through routine screening, using doppler ultrasound scanning. Anticoagulant therapy was immediately started and continued for three months, which resulted in resolution of the deep vein thrombosis. *Discussion*. To our knowledge, this is the first description of asymptomatic proximal DVT that was detected in a psychiatric inpatient setting. In light of the reported causal relationship between DVT and pulmonary embolism, screening for DVT can be of high clinical value in patients with psychiatric disorders, especially when their physical activity is highly compromised.

## 1. Introduction

Pulmonary embolism (PE) is a serious, life-threatening condition and most commonly derives from deep vein thrombosis (DVT) of the lower extremities [1]. Most DVTs originate in the calves, and 80% of distal DVTs are known to resolve spontaneously [1]. However, once DVTs reach a proximal vein (i.e., popilteal vein or higher), PE reportedly occurs in up to 50% of patients [1]. Therefore, in order to prevent PE, it is critically important to detect DVTs of the lower extremities.

DVTs have been reported to occur in up to 10–40% of hospitalized patients with a physical morbidity [2], and approximately 70–80% of such DVTs are asymptomatic [3]. Although there has been no systematic survey on the incidence of DVTs in psychiatric settings, it is very likely to be high, considering the lowered physical activity level of patients with psychiatric disorders. In our institution, we have therefore been conducting a routine screening of DVTs for inpatients who have been bedridden (i.e., deeply sedated or catatonic) for ≥2 days since October, 2009. In this screening, a D-dimer level is measured for all these patients when they are ambulant, and doppler ultrasound scanning is performed when the level is higher than 0.5 *μ*g/dL. 

 Here, we report on a patient with major depressive disorder in whom an asymptomatic proximal DVT was detected through routine screening. 

## 2. Case Presentation

A 65-year-old woman with a 13-year history of major depressive disorder was admitted to our hospital because of depressive mood, appetite loss, insomnia, and psychomotor retardation. She had no past history of any physical illness. Since she did not respond to paroxetine 40 mg/day or mirtazapine 45 mg/day, intravenous administration of clomipramine 25 mg/day was started on Day 7 with hydration of 1500 mL/day. However, she did not show any improvement, and she developed a catatonic state on Day 21. Since she had been laying on a bed all day without any voluntary movement, routine screening for DVTs was performed on Day 28; her plasma D-dimer level was elevated at 3.20 *μ*g/dL, and doppler ultrasound scanning revealed a 11 × 70 mm thrombosis in her left femoral vein. Anticoagulant therapy, consisting of warfarin 1 mg/day and subcutaneous injection of unfractionated heparin 10000 IU/day, was started, and the dose of warfarin was adjusted to achieve 2.0-3.0 in International Normalized Ratio. Warfarin was continued for three months, which resulted in resolution of the DVT. Her depressive symptoms were then successfully treated with electroconvulsive therapy. 

## 3. Discussion

To our knowledge, this is the first description of asymptomatic proximal DVT that was detected in a psychiatric inpatient setting. Catatonia is a common manifestation of psychiatric illnesses and characterized by a lack of voluntary physical activity. Considering that this patient did not have any other DVT risk factors, a lack of physical activity caused by catatonia would be expected to have triggered a development of DVT. Consistent with this, 22 cases of PE in catatonic patients were previously reported [4]. Several guidelines encouraged prophylactic interventions for the prevention of DVTs for hospitalized patients [5], which has not yet been addressed in psychiatric settings. Although there has been no systematic survey on the incidence of DVTs in psychiatric settings, it is likely to be high in patients with psychiatric illnesses, given their physical inactivity and the sedative effects of psychotropics. In fact, it was reported that antipsychotic usage was a risk factor for DVT [6]. Furthermore, approximately 70–80% of DVTs are clinically silent or asymptomatic [3], which underscores the need of screening for DVTs in high-risk patients. These findings suggest that screening for DVTs can be of high clinical value to effectively detect and treat DVTs in patients with psychiatric disorders, especially when their physical activity is highly compromised. 

## Abbreviations

DVT: Deep vein thrombosis
INR: International normalized ratio
PE: Pulmonary embolism.
